# Long-term extracorporeal membrane oxygenation - from SARS-CoV-2 infection to lung transplantation

**DOI:** 10.5935/0103-507X.20220314-en

**Published:** 2022

**Authors:** Mafalda Gama, Joana Cabrita, Cleide Barrigoto, Lúcia Proença, Philip Fortuna

**Affiliations:** 1 Unidade de Urgência Médica, Hospital de São José, Centro Hospitalar Universitário de Lisboa Central - Lisboa, Portugal

**Keywords:** COVID-19, Coronavirus infection, SARS-CoV-2, Respiratory distress syndrome, Extracorporeal membrane oxygenation, Lung transplantation

## Abstract

A healthy 55-year-old woman unvaccinated for SARS-CoV-2 was admitted to the hospital with a SARS-CoV-2 infection with rapid clinical deterioration. On the 17th day of disease, she was intubated, and on the 24th day, the patient was referred and admitted to our extracorporeal membrane oxygenation center. Extracorporeal membrane oxygenation support was initially used to enable lung recovery and allow the patient to rehabilitate and improve her physical condition. Despite an adequate physical condition, the lung function was not adequate to discontinue extracorporeal membrane oxygenation, and the patient was considered for lung transplantation. The intensive rehabilitation program was implemented to improve and maintain the physical status throughout all phases. The extracorporeal membrane oxygenation run had several complications that hindered successful rehabilitation: right ventricular failure that required venoarterial-venous extracorporeal membrane oxygenation for 10 days; six nosocomial infections, four with progression to septic shock; and knee hemarthrosis. To reduce the risk of infection, invasive devices (i.e., invasive mechanical ventilation, central venous catheter, and vesical catheter) were removed whenever possible, keeping only those essential for monitoring and care. After 162 days of extracorporeal membrane oxygenation support without other organ dysfunction, bilateral lobar lung transplantation was performed. Physical and respiratory rehabilitation were continued to promote independence in daily life activities. Four months after surgery, the patient was discharged.

## INTRODUCTION

Acute respiratory distress syndrome (ARDS) that requires mechanical ventilation occurs in 10% of patients with severe acute respiratory syndrome coronavirus 2 (SARS-CoV-2) infection.^(1,2)^ Some patients have treatment-refractory ARDS and need respiratory extracorporeal membrane oxygenation (ECMO) support. Lung transplantation is accessible to a few selected patients with irreversible lung disease who fulfil all the clinical criteria.^(3,4)^

There are no stringent indications for lung transplantation in the context of coronavirus disease 2019 (COVID-19); nonetheless, there are some recommendations based on accumulated experience. The following criteria are recommended to qualify candidates for lung transplantation: 65 years of age or younger, have single organ dysfunction, sufficient time to allow for lung recovery (lung transplantation is recommended only after four to six weeks of initial clinical signs of respiratory failure), radiological evidence of irreversible lung disease (severe bullous destruction or evidence of established fibrosis), be in a conscious state to discuss transplantation and participate in physical rehabilitation, fulfil the remaining typical criteria for transplantation (adequate body mass index, absence of other notable comorbidities such as coronary artery disease or cancer) and have a recent negative result for SARS-CoV-2 by polymerase chain reaction (PCR).^(5)^

## CASE REPORT

A healthy 55-year-old woman unvaccinated for SARS-CoV-2, known to be positive for SARS-CoV-2 for nine days, was admitted to the emergency department of another hospital with a 10-day history of fever, cough, and dyspnea.

In the medical ward, dexamethasone 6 mg was started and maintained for 10 days. Rapid clinical deterioration was observed. On the 13th day of symptoms, the patient developed severe hypoxemia (partial arterial oxygen pressure 66mmHg on 15L/minute of oxygen by high concentration mask), and she was admitted to the intensive care unit (ICU). Initially, the patient presented an adequate response to high-flow oxygen and a self-prone position; however, on the 17th day, her condition deteriorated further, and she was invasively ventilated. Despite prone positioning, neuromuscular blockade, positive end-expiratory pressure titration under transthoracic impedance guidance, 100% fraction of inspired oxygen and high mechanical power ventilation, refractory hypoxemia and low lung compliance (38mL/cmH_2_O) persisted. Late ventilatory-associated pneumonia (VAP) due to *Acinetobacter baumannii* was considered a contributor to clinical impairment. Meropenem was administered initially and later changed to trimethoprim/sulfamethoxazole according to the antibiotic susceptibility tests. On day 25 of the disease, she was referenced and accepted to our ECMO center.

An ECMO team met the patient at the local hospital and performed percutaneous cannulation of the right common femoral vein (Getinge Maquet®, 23F, 55cm) and right internal jugular vein (Getinge Maquet®, 19F, 15cm) under ultrasound guidance. Venovenous ECMO (VV-ECMO) was started, and the patient was transferred to our ECMO center. Anticoagulation with nonfractional heparin was initiated and adjusted per the protocol.

The ECMO run lasted 162 days and can be divided into two major periods: the acute phase of critical illness and the chronic phase of critical illness.

In the acute phase of critical illness, the main goal was to provide the best conditions for lung recovery.

Initially, ECMO support was increased due to respiratory worsening and persistent polypnea despite analgosedation titration and neuromuscular blockade. Hyperactive *delirium* was also managed.

On the 35th day of the disease, a thoracic computed tomography (CT) scan revealed organizing pneumonia. Thus, high-dose corticosteroid therapy was administered (1g/day for three days, 1mg/kg/day for 15 days followed by progressive weaning).

Physical and respiratory rehabilitation was implemented as part of the strategy for lung recovery and myopathic prevention. Percutaneous tracheostomy was performed after 25 days of endotracheal intubation.

On the 36th day of ECMO, venoarterial-venous ECMO (VAV-ECMO) reconfiguration was implemented due to right ventricular failure and septic shock. Multirresistant *Staphylococcus epidermidis* bacteremia and VAP to *Klebsiella oxytoca* ESBL+ were detected. For this purpose, the left common femoral artery was cannulated (Getinge Maquet®, 17F, 23cm). For distal limb perfusion, the ipsilateral superficial femoral artery was cannulated (CruraSave®, 8F). The patient was on VAV-ECMO for 10 days. After partial reversal of cardiac impairment, the patient was decannulated from venoarterial ECMO (VA-ECMO) without complications. The access site of the common femoral artery was closed with a percutaneous vascular closure device (Teleflex© Manta® device), and pressure was applied on the access site of the distal perfusion cannula. Respiratory support was maintained through VV-ECMO.

Later, a pseudoaneurysm of the left superficial femoral artery (on the distal perfusion access site) was diagnosed, and the rehabilitation program had to be discontinued for 10 days. Three weeks later, the pseudoaneurysm was repaired with a thrombin injection.

After two months of ECMO support with no respiratory improvement, a thoracic CT scan showed a right basal cavitation of 65 x 46mm. To decrease the iatrogenic risk, mechanical ventilation was substituted for high flow oxygen (through tracheostomy). Stenotrophomonas maltophilia was isolated in the bronchoalveolar lavage.

The chronic phase of critical illness lasted approximately three months. Both the recovery and transplant phase should necessarily be supported by rehabilitation. An intensive rehabilitation program was applied to improve and maintain the physical status.

Invasive devices were maintained only for essential monitoring and care. Ventilation through tracheostomy was performed for 76 days with progressive weaning. After tracheostomy decannulation, she was on binasal high-flow oxygen and then on binasal cannula oxygen therapy. The central venous catheter and vesical catheter were removed; thus, intravenous therapy was administered through the ECMO circuit, and fluid balance was managed by weight surveillance.

Once again, interruption of rehabilitation occurred due to spontaneous knee hemarthrosis, a hemorrhagic complication of anticoagulation therapy.

During the ECMO run, she had six nosocomial infections, two of which were respiratory infections. Four of these infections progressed to septic shock, requiring a total of 91 days of antibiotic therapy. All infections were adequately treated.

The patient participated in intensive physical rehabilitation, until eventually the patient was able to walk through the ICU under ECMO support.

**Figure 1:**
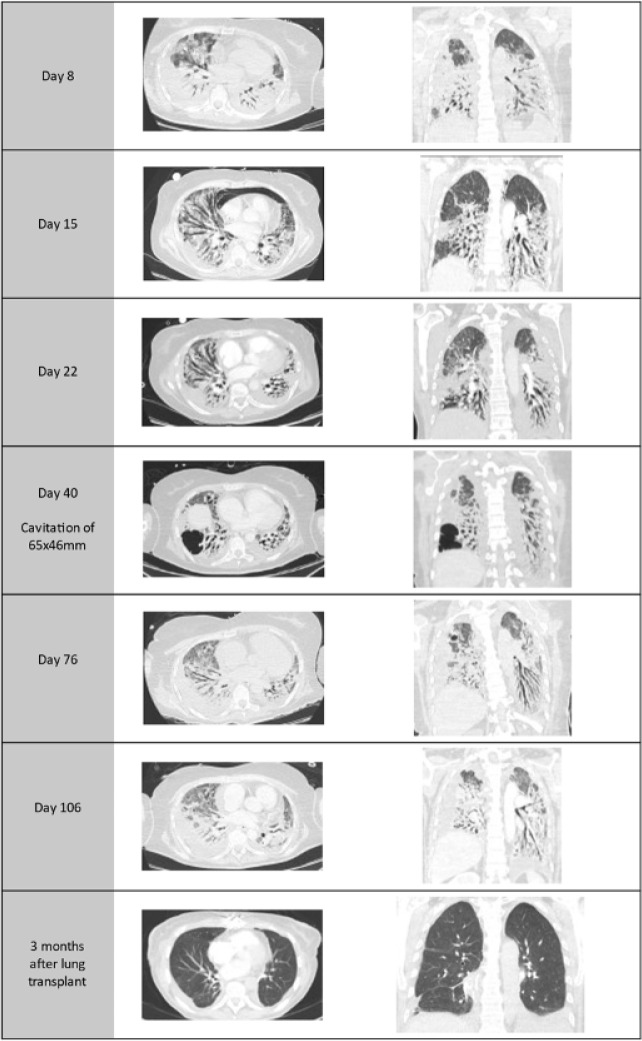
Serial thoracic computed tomography scan evaluations (days are counted starting with the initiation of extracorporeal membrane oxygenation).

After serial CT scan evaluations (Figure 1), good physical rehabilitation and clinical assessment, with numerous failed attempts to stop ECMO support, an irreversible lung injury with total pulmonary ECMO support dependence was established. The patient presented some favorable factors that made her a candidate for lung transplantation, such as age, single organ dysfunction, physical rehabilitation adherence and not having any general exclusion criteria for transplantation. Therefore, the patient was proposed and accepted for a lung transplant.

**Figure 2:**
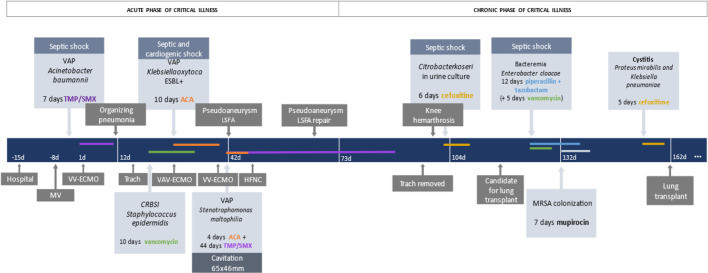
Timeline before and during the extracorporeal membrane oxygenation run.

The patient was on ECMO support for 162 days with almost half of this time as a bridging strategy for lung transplantation (Figure 2). The access cannula and the venous return cannula were the same throughout the ECMO run. On the transplantation day, the patient was on 2L/minute oxygen by a binasal cannula and VV-ECMO support (blood flow of 3,4L/minute and sweep gas flow of 3L/minute). There was no other organ dysfunction.

VAP - ventilatory associated pneumonia; TMP/SMX - trimethoprim/sulfamethoxazole; ESBL - *extended-spectrum beta-lactamases*, ACA - amoxicillin/clavulanic acid; LSFA - left superficial femoral artery; MV - mechanical ventilation; VV-ECMO - venovenous extracorporeal membrane oxygenation; Trach - tracheostomy; VAV-ECMO - venoarterial-venous extracorporeal membrane oxygenation; HFNC - high-flow nasal cannula; CRBSI - catheter-related bloodstream infection; MRSA - methicillin-resistant *Staphylococcus aureus.*

Bilateral lobar lung transplantation was performed under VA-ECMO. The postoperative period was hampered by *delirium*. Once again, she was tracheostomized for approximately one month. After 22 days of postoperative ICU stay, she was admitted to the Pulmonology ward under low concentration oxygen therapy. As an immunocompromised patient, she had several complications: cystitis due *to Proteus mirabilis*, pneumonia due to methicillin-susceptible *Staphylococcus aureus* and *Enterobacter cloacae*, neutropenia, and asymptomatic minimal acute rejection of the graft (grade A1). Physical and respiratory rehabilitation were maintained to improve autonomy in daily life activities. Four months after surgery, the patient was discharged without oxygen supplementation, and no complications have been reported to date.

## DISCUSSION

Lung transplantation after COVID-19-related persistent lung injury is rare (fewer than 100 cases reported). To our knowledge, this is the second longest ECMO run of a COVID-19 patient with irreversible lung disease until successful lung transplantation. The patient who had the longest ECMO run died nine months after lung transplantation due to acute rejection of the graft.^(6)^

ECMO was primarily used as a bridge to recovery; after irreversible lung injury was established, VV-ECMO was employed to guarantee complete respiratory support and enable physical rehabilitation. The patient did not have any exclusion criteria for lung transplantation, as a good performance status was achieved and maintained.

COVID-19 is still a new disease; thus, clinical and radiological criteria for irreversible lung injury have yet to be defined.^(7)^ In this case, irreversible lung injury was established after serial CT scans showing persistent lung fibrosis after two months of protective mechanical ventilation, long-term corticosteroid therapy, intensive physical rehabilitation and complete VV-ECMO dependency.

The patient suffered many complications in the ICU that were contributors to recessions in physical rehabilitation, including various nosocomial infections and hemorrhagic complications. Intensive care unit length of stay, exposure to multiple invasive devices and immunosuppression induced by critical illness and corticosteroid therapy were contributing factors in the number of nosocomial infections. Therapy simplification and device removal are essential to decrease the risk of infection and improve rehabilitation.

## CONCLUSION

Venovenous extracorporeal membrane oxygenation is an extracorporeal support device that guarantees total lung support in cases of respiratory failure. It has been increasingly used as a bridge to transplant in patients with terminal lung disease who would otherwise not survive until transplantation. In COVID-19 patients, venovenous extracorporeal membrane oxygenation has been used with good outcomes, but it is only rarely kept as a bridge for lung transplantation when irreversible lung injury is established.

Although more common since the COVID-19 pandemic, venovenous extracorporeal membrane oxygenation still has risks and should be performed in centers with high expertise and volume to improve care and outcome in patients under extracorporeal membrane oxygenation support, mostly when a long run is needed. A multidisciplinary team is vital to select possible candidates for lung transplantation, implement a rehabilitation program and reduce the risks of long extracorporeal membrane oxygenation support and intensive care unit stay.

With this clinical report, we intend to show that lung transplantation is a treatment solution for COVID-19 patients with persistent lung injury when venovenous extracorporeal membrane oxygenation is used as a bridge to transplantation. Additionally, rehabilitation is the cornerstone for lung recovery, physical conditioning and success of lung transplantation.

### Statement of ethics

Our institution does not require ethical approval for reporting individual cases. Written informed consent was obtained from the patient for publication of this case report.
